# Exposure of a Distinct PDCA-1^+^ (CD317) B Cell Population to Agonistic Anti-4-1BB (CD137) Inhibits T and B Cell Responses Both In Vitro and In Vivo

**DOI:** 10.1371/journal.pone.0050272

**Published:** 2012-11-21

**Authors:** Dass S. Vinay, Seung J. Lee, Chang H. Kim, Ho Sik Oh, Byoung S. Kwon

**Affiliations:** 1 Section of Clinical Immunology, Allergy, and Rheumatology, Department of Medicine, Tulane University Health Sciences Center, New Orleans, Louisiana, United States of America; 2 Cell and Immunobiology, and R and D Center for Cancer Therapeutics, National Cancer Center, Goyang-Si, Gyeonggi-Do, Korea; Beth Israel Deaconess Medical Center, Harvard Medical School, United States of America

## Abstract

4-1BB (CD137) is an important T cell activating molecule. Here we report that it also promotes development of a distinct B cell subpopulation co-expressing PDCA-1. 4-1BB is expressed constitutively, and its expression is increased when PDCA-1^+^ B cells are activated. We found that despite a high level of surface expression of 4-1BB on PDCA-1^+^ B cells, treatment of these cells with agonistic anti-4-1BB mAb stimulated the expression of only a few activation markers (B7-2, MHC II, PD-L2), cytokines (IL-12p40/p70), and chemokines (MCP-1, RANTES), as well as sTNFR1, and the immunosuppressive enzyme, IDO. Although the PDCA-1^+^ B cells stimulated by anti-4-1BB expressed MHC II at high levels and took up antigens efficiently, Ig class switching was inhibited when they were pulsed with T-independent (TI) or T-dependent (TD) Ags and adoptively transferred into syngeneic recipients. Furthermore, when anti-4-1BB-treated PDCA-1^+^ B cells were pulsed with OVA peptide and combined with Vα2^+^CD4^+^ T cells, Ag-specific cell division was inhibited both in vitro and in vivo. Our findings suggest that the 4-1BB signal transforms PDCA-1^+^ B cells into propagators of negative immune regulation, and establish an important role for 4-1BB in PDCA-1^+^ B cell development and function.

## Introduction

4-1BB (TNFRSF9; CD137) is a 45–50 kDa protein that is expressed constitutively by CD4^+^Foxp3^+^ T regulatory (Treg) and CD11c^+^ dendritic cells (DCs) and by T, NK, and NKT cells, mainly when they are activated [1]–[5]. In vitro 4-1BB signals stimulate both CD4^+^ and CD8^+^ T cells to a similar extent, resulting in enhanced cell division, upregulation of cell survival genes, induction of cytokines, and prevention of activation-induced cell death [6]. Interestingly, in vivo administration of agonistic anti-4-1BB results in a biased CD8^+^ T cell response with a concomitant decline of NK, CD4^+^ T, and B cell numbers and functions [2], [3], [7], [8]. This strong ability of anti-4-1BB to amplify CD8^+^ T cells in vivo has emerged as a valuable therapeutic tool to counter bacterial and viral infection, cancer, transplant rejection, graft-versus-host disease, and autoimmune disease [2], [3], [7], [8]. The precise mechanism of the skewed CD8^+^ T cell response to anti-4-1BB in vivo is not fully understood, but several of the molecules involved have been identified; increased levels of interferon (IFN)-γ [8]–[10], tumor necrosis factor (TNF)-α [8], transforming growth factor (TGF)-β [11], [12], and indoleamine 2,3-dioxygenase (IDO) [13], [14] play key roles.

Although the consequences of 4-1BB signaling have been extensively investigated in T, NK, and NK T cells [2], [3], [7], [8], this is not the case for non-T cells. Investigation of 4-1BB signaling in these cells is important, as functional 4-1BB has been found on a number of non-T cells, including DCs, monocytes, B cells, neutrophils, and mast cells, both under physiological conditions and in situations involving disease-induced inflammation [15].

Plasmacytoid dendritic cells (pDCs) are an important class of immune regulators that play a central role in anti-viral immunity, mainly via their production of type I interferons (IFNs) [16]. Mouse pDCs have been found in lymphoid organs, liver, lung, heart, blood vessels, and skin [17], [18]. Human pDCs populate primary, secondary and tertiary lymphoid organs, the liver, and the blood [19]. Mouse pDCs share most morphological and phenotypic features with their human counterparts; however, they are defined as CD11c^+^PDCA-1^+^Gr1^+^B220^+^120G8^+^ cells [17], [20] while human pDCs are BDCA-2/4^+^CD4^+^CD45RA^+^IL-3αR^+^ (CD123) ILT3^+^ILT1^−^CD11c^low/−^ [20]. Although PDCA-1 is a signature marker of pDCs [20], many cell types express this antigen when activated, including B lymphocytes [20]. In pathological conditions, pDCs migrate from the bone marrow (BM) to damaged tissue through high endothelial venules [19]. Elimination of pDCs with depleting Abs has been shown to have important effects on immune regulation [21]–[23].

In this study we found that 4-1BB is expressed constitutively on a distinct PDCA-1^+^ B cell population, and is upregulated further upon activation. A recent study revealed functional 4-1BB expression on human B cells [24]. However, we observed that, conv B (PDCA-1^−^CD19^+^IgD^+^) cells or conv pDCs (i.e. PDCA-1^+^CD19^−^IgD^−^) express little or no 4-1BB under physiological conditions, and expression is only modestly increased upon activation in our mouse studies. Furthermore, exposure of PDCA-1^+^ B cells to agonistic anti-4-1BB was found to have negative immune regulatory effects both in vitro and in vivo. Thus, our observations have revealed a hitherto unknown facet of 4-1BB signaling, namely as an important regulator of PDCA-1^+^ B cell development and function.

## Results

### PDCA-1^+^ B cells constitutively express 4-1BB

**Figure 1:**
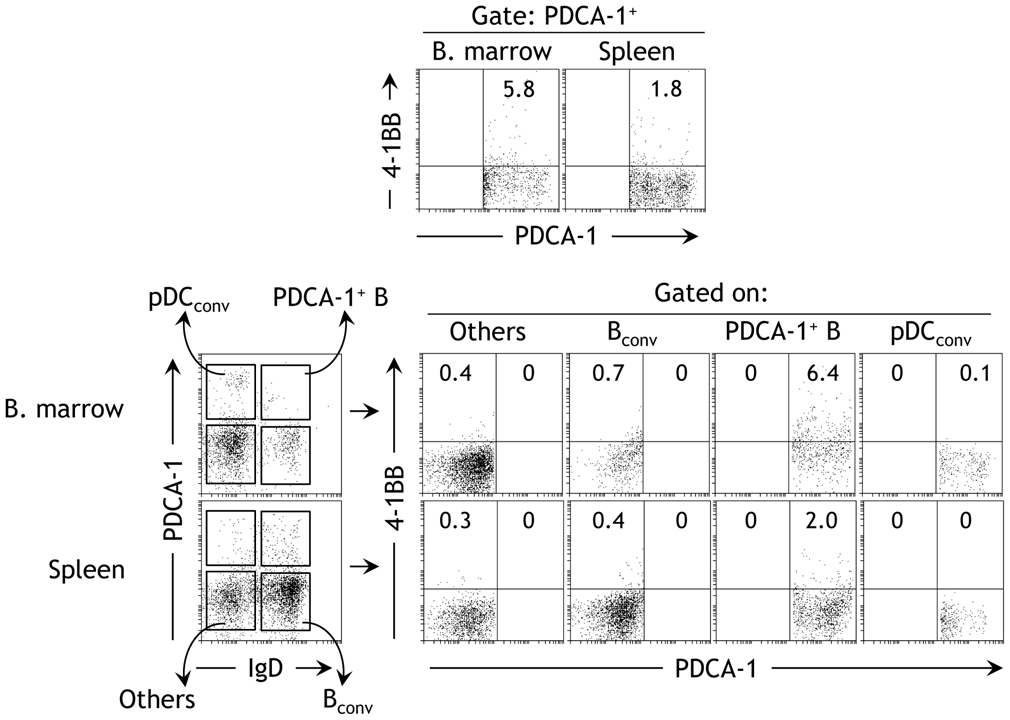
PDCA-1^+^ B cells constitutively express 4-1BB. Bone marrow (BM)-derived and spleen cells from naïve B6 mice were subjected to flow cytometry with the indicated markers. **A** The proportions of 4-1BB^+^ cells in PDCA-1^+^-gated populations are indicated. One of six independent experiments is shown (*n* = 6). **B** The different populations of cells from BM and spleen are indicated. One of three independent experiments is shown (*n* = 3). **C** Gates were set around the indicated cell populations. The proportion of 4-1BB-expressing cells in each population is indicated. One of three independent experiments is shown (*n* = 3).

We found that PDCA-1^+^ cells constitutively express 4-1BB in naïve mice (Fig. 1A). The expression was higher in the bone marrow (Fig. 1A left panel) than in the spleen (Fig. 1A right panel). We found previously that PDCA-1^+^ cells in naïve mice consist of at least two subsets; DC-derived pDCs, and a rare functional B cell subpopulation [25]. Therefore, we next determined to which of these populations the observed PDCA-1^+^ cells expressing 4-1BB belonged. To this end, we stained cells with a B cell-specific B cell marker, IgD, to distinguish B cells from non-B cells (Fig. 1B), and examined the expression of 4-1BB within the gated cell populations. We found that constitutive expression of 4-1BB was found only within the PDCA-1^+^IgD^+^ subpopulation of both spleen and bone marrow (Fig. 1C). A similar expression was also observed on purified CD19^+^ B cells obtained from the spleens and bone marrow of naïve mice (data not shown). In addition, the PDCA^+^CD19^+^4-1BB^+^ cells are also found in the peritoneal cavity, blood (of both mice and humans), and livers (data not shown). Other cell fractions displayed little or no 4-1BB expression, suggesting that in naïve mice 4-1BB expression in PDCA-1^+^ cells is restricted to PDCA-1^+^IgD^+^ B cells (hereafter referred to as PDCA-1^+^ B cells).

### Activation upregulates 4-1BB expression in PDCA-1^+^ cells

**Figure 2:**
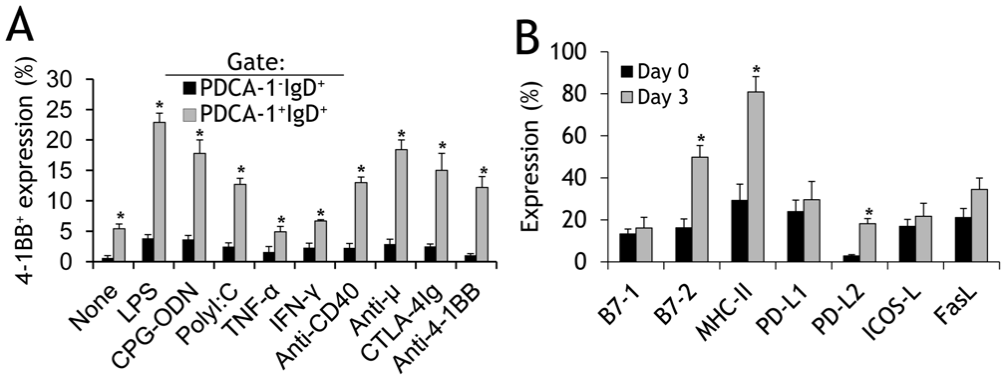
Activation upregulates 4-1BB expression in PDCA-1^+^ cells. **A** Total BM-derived cells were stimulated with the indicated soluble agonists for 3d, stained with fluorochrome- tagged Abs to 4-1BB, PDCA-1, and IgD, then analysed by flow cytometry. The percentages of 4-1BB^+^ cells in gated PDCA-1^+^IgD^+^ and PDCA-1^+^IgD^−^ fractions are presented as bar graphs (mean ± SD). The results of three pooled independent experiments are shown (*n* = 3). **B** Purified splenic PDCA-1^+^ B cells from naïve B6 mice were stimulated with anti-4-1BB (5 µg/ml) for 3d, stained as indicated, and flow cytometry was performed. The frequencies of cells expressing the indicated activation markers were evaluated and are presented as bar graphs (mean ± SD). The results of three pooled independent experiments are shown (*n* = 3). * *p*<0.05.

After determining that PDCA-1^+^ B cells but not conv B cells (PDCA-1^−^IgD^+^) cells constitutively express 4-1BB, we next investigated whether activation upregulates 4-1BB expression in these subpopulations. When bone marrow–derived cells were stimulated, we found that expression of 4-1BB was significantly upregulated on both PDCA-1^+^ B cells and on conv B cells (PDCA-1^−^IgD^+^) by almost all the agonists tested (Fig. 2A). Although 4-1BB expression on activated conv B cells was several-fold higher than the 0.6% expression on non-activated conv B cells, the maximum expression still did not exceed 5% (Fig. 2A; black bars). In contrast, expression of 4-1BB on PDCA-1^+^ B cells increased from a basal value of 5.4% to over 20% (Fig. 2A, grey bars). Interestingly, stimulation with anti-μ (a B cell-specific agonist) significantly upregulated 4-1BB expression on PDCA-1^+^ B cells but only moderately on conv B cells (Fig. 2A). We also found that activation with agonistic anti-4-1BB upregulates 4-1BB expression more on PDCA-1^+^ B cells than on conv B cells (Fig. 2A), suggesting that the 4-1BB on PDCA-1^+^ B cells is functional. After discovering that signaling by 4-1BB upregulates its own expression, we purified PDCA-1^+^CD43^−^ B cells, stimulated them with agonistic anti-4-1BB mAb, and evaluated the expression of various activation markers. We found that only B7-2, MHC-II, and PD-L2 were significantly upregulated (Fig. 2B).

### 4-1BB signaling promotes PDCA-1^+^ B cell development

**Figure 3:**
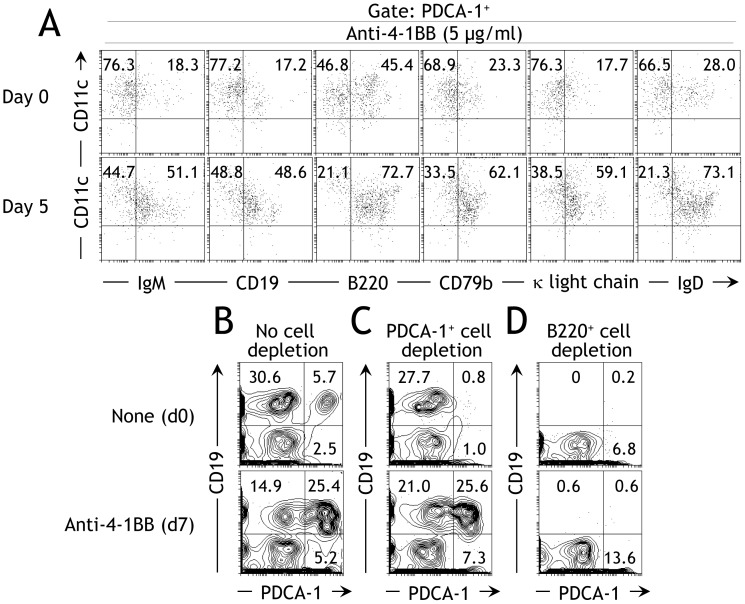
4-1BB signaling promotes PDCA-1^+^ B cell development. **A** BM-derived cells from naïve B6 mice were stained with the indicated markers before (upper panels) or after 5d (lower panels) activation with soluble anti-4-1BB (5 µg/ml) and flow cytometry was performed. The frequencies of CD19^+^CD11c^+^ cells, among gated PDCA-1^+^ cells, are indicated. One of three independent experiments is shown (*n* = 3). **B**–D BM-derived cells from naïve B6 mice were stained for the indicated markers either with no cell depletion **B**, after PDCA-1^+^ cell depletion **C**, or after B220^+^ cell depletion **D**. The cells were stained either immediately (upper row) or after 7d stimulation with soluble anti-4-1BB (5 µg/ml) (lower row). One of two independent experiments is shown (*n* = 2).

As our data indicated that signaling via 4-1BB activated PDCA-1^+^ B cells (Fig. 2), we next investigated whether the anti-4-1BB promotes the development of PDCA-1^+^ B cells. When bone marrow-derived cells were stimulated with anti-4-1BB for 5d, we found a 2–3-fold increase in the proportion of PDCA-1^+^ B cells as identified by the co-expression of several B cell-specific molecules (Fig. 3A). Interestingly, there was a 2–3-fold reciprocal reduction in the proportion of conv pDCs (PDCA-1^+^CD11c^+^ and no expression of B cell-specific molecules). These results confirm that 4-1BB signaling promotes development of PDCA-1^+^ B cells from bone marrow. After finding that 4-1BB signaling promotes development of PDCA-1^+^ B cells from bone marrow, we next tested whether these cells were derived from existing PDCA-1^+^ B cells or from a precursor population. To investigate this we used agonistic anti-4-1BB mAb to stimulate BM-derived cells, with either no cell depletion, or with depletion of PDCA-1^+^ cells or B220^+^ cells using anti-PDCA-1^+^ and B220^+^ microbeads, respectively. When stimulated for 7d with anti-4-1BB, the undepleted population displayed a 2-fold decrease in the proportion of conv B cells (PDCA-1^−^CD19^+^) but a 2-fold increase in the proportion of conv pDCs (PDCA-1^+^CD19^−^) and a 5-fold increase in the proportions of PDCA-1^+^CD19^+^ B cells (Fig. 3B; compare upper and lower panels). Interestingly, depletion of existing PDCA-1^+^ cells did not prevent the development of PDCA-1^+^ B cells (Fig. 3C; compare upper and lower panels). However, the proportion of conv B cells remained unperturbed in these cultures after anti-4-1BB stimulation (Fig. 3C; compare upper and lower panels). On the other hand, depletion of B220^+^ cells completely prevented development of PDCA-1^+^ B cells (Fig. 3D; compare upper and lower panels). Taken together, these data suggest that 4-1BB signaling does not promote development of PDCA-1^+^ B cells from conv pDCs or existing PDCA-1^+^ B cells but rather from a B cell precursor population.

### 4-1BB signaling induces expression of IDO, no type I IFN, and limited cytokines in PDCA-1^+^ B cells

**Figure 4:**
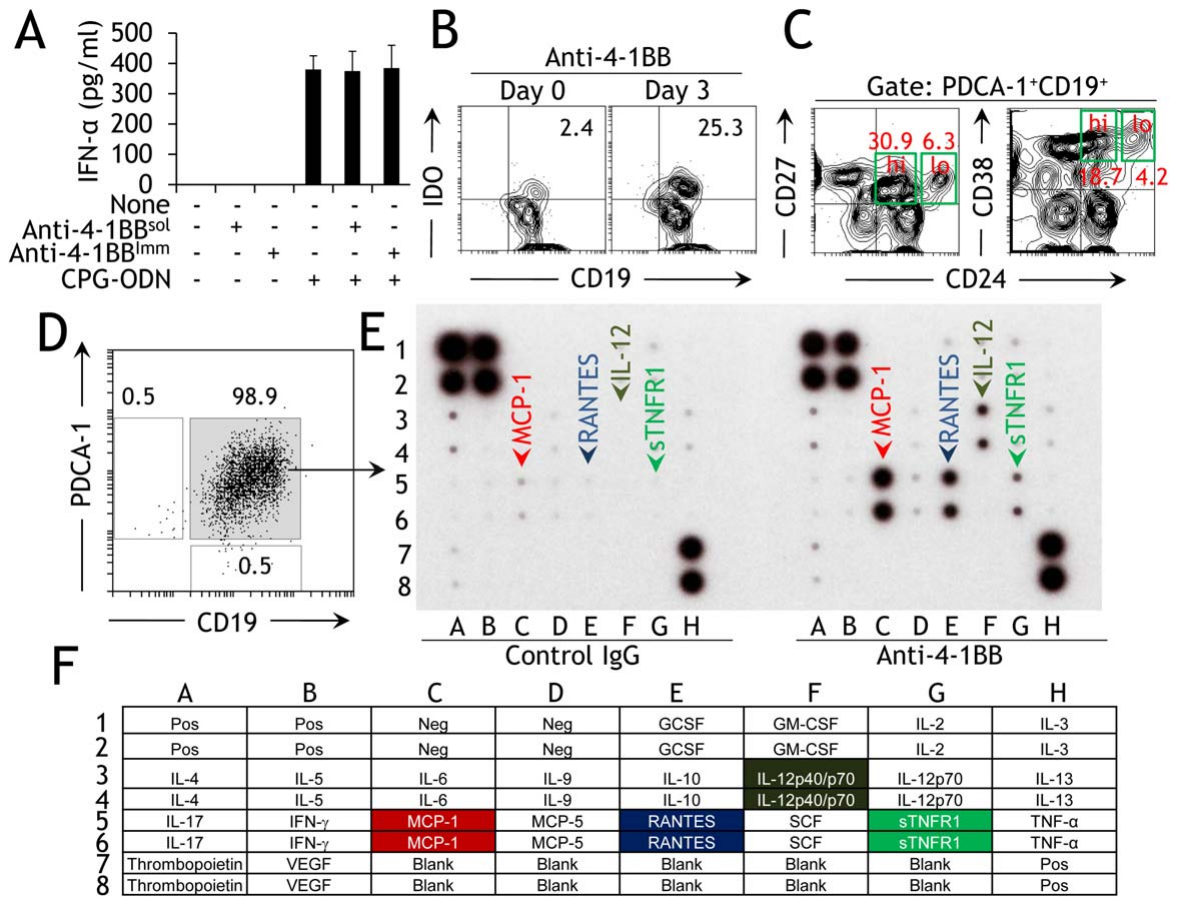
4-1BB signaling induces selective expression of effector molecules. **A** Purified splenic PDCA-1^+^ B cells from naïve B6 mice were stimulated with soluble anti-4-1BB (5 µg/ml) for 3d, cell-free culture supernatants were collected, and levels of IFN-α were analyzed by ELISA. These values are presented as bar graphs (mean ± SD). The results of three pooled independent experiments are shown (*n* = 2). **C** Total bone marrow-derived cells were stimulated with anti-4-1BB (5 µg/ml). After 6d, cells were collected, washed, stained as indicated, and analyzed. One of three independent experiments is shown (*n* = 3). **D** Purified splenic PDCA-1^+^ B cells were stained for the indicated markers either immediately or 3d after stimulation with anti-4-1BB (5 µg/ml). One of two independent experiments is shown (*n* = 3). **C** FACS-sorted PDCA-1^+^ B cells from spleens of naïve mice were stained as indicated and flow cytometry was performed. One of three independent experiments is shown (*n* = 3). **E** Sorted PDCA-1^+^ B cells were stimulated for 3d with rat IgG or soluble anti-4-1BB (both 5 µg/ml), and cell-free supernatants were collected and analyzed for cytokines using commercially available array screens. The results of three pooled independent experiments are shown (*n* = 3). **F** Table showing the position of individual cytokines/chemokines used in the assay. Upregulated molecules in the cells stimulated with anti-4-1BB and their position in the array are highlighted. Pos, positive; Neg, negative.

pDCs are defined as professional interferon (IFN)-producing cells (IPCs), and their exposure to viruses, bacteria, or specific synthetic TLR agonists can lead to production of, among others, type I IFNs [26]. Activated PDCA-1^+^ B cells also secrete type I IFNs [25], [27]. We therefore investigated whether 4-1BB induced IFN-α production in these cells. We found that stimulation of PDCA-1^+^ B cells with agonistic anti-4-1BB mAb (either soluble [sol] or immobilized [imm]) did not induce IFN-α expression (Fig. 4A). To test if 4-1BB signaling in PDCA-1^+^ B cells inhibits IFN-α expression, we treated cultures with both anti-4-1BB and TLR9 agonist, a known inducer of type I IFNs [16]. There was no effect of anti-4-1BB on the amount of CPG-ODN-mediated IFN-α produced in these cultures (Fig. 4A). These data indicate that 4-1BB signaling, although important for the activation and development of PDCA-1^+^ B cells, is not required for induction of type I IFNs in these cells.

Since expression of IDO has been identified as a prominent feature of activated pDCs [28], and our previous studies showed that PDCA-1^+^ B cells can be induced to express IDO [25], we next evaluated expression of this enzyme. Flow cytometric analysis of purified PDCA-1^+^ B cells showed that naïve cells expressed only basal levels of IDO which was upregulated 10-fold upon activation with anti-4-1BB (Fig. 4B). We have further characterized the PDCA^+^4-1BB^+^ B cells to determine if they shared phenotypes with other reported regulatory B cells (Bregs) [29], [30]. Our results show that the anti-4-1BB activated PDCA^+^ B cells co-express CD24^hi^CD27 or CD24^hi^CD38^hi^ (molecules expressed on certain Bregs; [29], [30]) only minimally compared to the CD24^lo^CD27 or CD24^lo^CD38^lo^ which was expressed 4–5-fold fold higher on these cells (Fig. 4C). After finding that 4-1BB signaling did not lead to type I IFN production in PDCA-1^+^ B cells, we next examined whether anti-4-1BB induced expression of other cytokines in these cells. FACS-sorted splenic PDCA-1^+^ B cells were collected (Fig. 4D) and incubated for 3d with anti-4-1BB or rat IgG as a negative control, and cell-free supernatants were analyzed using commercially available cytokine array screens. We found that of the 23 cytokines assayed, only MCP-1, RANTES, IL12p40/p70, and sTNFR1 (soluble TNFR1) were upregulated in anti-4-1BB supplemented cultures compared with IgG supplemented cultures (Fig. 4E and 4F). These data demonstrate that 4-1BB signaling promotes production of only a few specific cytokines, consistent with the observed limited upregulation of activation markers (Fig. 2B).

### Anti-4-1BB-preconditioned PDCA-1^+^ B cells inhibit Ag-specific T cell responses

**Figure 5:**
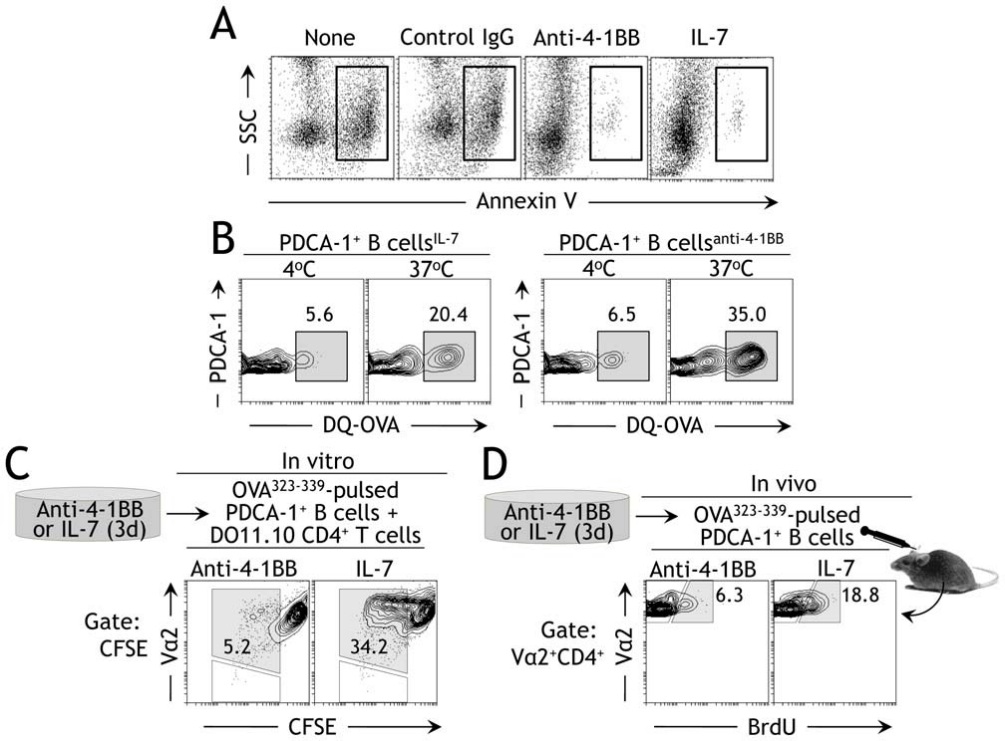
Anti-4-1BB-activated PDCA-1^+^ B cells inhibit Ag-specific T cell responses. **A** Purified PDCA^+^ B cells were treated for 3d with indicated markers and Annexin V assay was performed as depicted in Materials and methods. The events in the boxes represent cells undergoing apoptosis. None, no stimulation for 72 hrs. One of 2 independent experiments is shown (*n* = 2). **B** Purified PDCA^+^ B cells from the bone marrows of naïve Balb/C mice were stimulated for 3d with IL-7 (100 ng/ml) or soluble anti-4-1BB (5 µg/ml) and washed, and Ag uptake assays were performed as described in *Materials and Methods*. The cells were stained as indicated and flow cytometry was performed. One of two independent experiments is shown. *n* = 2. **C** Purified PDCA-1^+^ B cells were stimulated for 3d with IL-7 (100 ng/ml) or soluble anti-4-1BB (5 µg/ml), pulsed with 0.5 mg OVA^323–339^ peptide, and incubated with purified CFSE-labeled DO11.10 CD4^+^ T cells for 3d. They were stained as indicated and flow cytometry was performed. One of two independent experiments is shown. *n* = 2. Gates were set around CFSE^+^ cells, and proportions of Vα2^+^ cells were enumerated. **D** Purified PDCA-1^+^ B cells from naïve Balb/C mice were pulsed with OVA^323–339^ as in experiment **B**, and adoptively transferred (2×10^6^/mouse) into syngeneic DO11.10 mice. The recipient DO11.10 mice were further treated with BrdU as described in *Materials and Methods*. Spleen cells from the treated mice were prepared 3d after cell transfer, stained as indicated, and flow cytometry was performed. One of two independent experiments is shown. Gates were set around Vα2^+^CD4^+^ cells, and Vα2^+^BrdU^+^ cells were enumerated.

Although ∼95% of anti-4-1BB stimulated PDCA-1^+^ B cells expressed MHC II (Fig. 2B), it was not clear if they were capable of Ag uptake and could perform antigen presenting cell (APC) functions. To investigate this, sorted splenic PDCA-1^+^ B cells were incubated with anti-4-1BB or IL-7 for 3d. It may be mentioned here that the rat IgG could not be used as a control for anti-4-1BB group, in this and other experiments, mainly because of increased cell death/apoptosis in this group when stimulated for the same duration (Fig. 5A). The inclusion of IL-7 in this and other experiments was only to keep cells viable for the duration and does not represent an accurate istoype control for the anti-4-1BB group. The activated cells were then pulsed with fluorescently-labeled DQ-OVA at 4°C or 37°C to analyze their Ag uptake capability. PDCA-1^+^ B cells pre-activated with anti-4-1BB demonstrated Ag uptake at 37°C on a par with IL-7-stimulated cells (Fig. 5B). The specificity of the experiment was confirmed by conducting the Ag uptake assay under unfavorable conditions (i.e. 4°C) where background Ag uptake could be detected (Fig. 5B). After confirming that anti-4-1BB-pre-activated PDCA-1^+^ B cells can take up Ag normally, we tested if they could partner with T cells in a cognate manner to present Ag and induce Ag-specific signals in the T cells. To this end, we sort-purified splenic PDCA-1^+^ B cells and cultured them in the presence of anti-4-1BB for 3d. We used IL-7 as a positive control as it induced comparable Ag uptake and is known to have a role in supporting B cell growth [31]–[33]. Following incubation with anti-4-1BB or IL-7, the cells were pulsed with OVA^323–339^ and co-cultured for 3d with CFSE-labeled CD4^+^ T cells obtained from DO11.10 mice. The PDCA-1^+^ B cells cultured with IL-7 induced Ag-specific cell division in their Vα2^+^ T cell partners (Fig. 5C; right panel), whereas those cultured with anti-4-1BB failed to induce this cell division, although the non-divided Vα2^+^ cells could still be seen (Fig. 5C; left panel). When the OVA^323–339^-pulsed IL-7- or anti-4-1BB-pre-activated PDCA-1^+^ B cells were adoptively transferred into syngeneic DO11.10 recipients, the PDCA-1^+^ B cells treated with IL-7 induced Ag-specific Vα2^+^CD4^+^ T cell division, as judged by BrdU incorporation assays, whereas those treated with anti-4-1BB did not (Fig. 5D; compare panels left and right). These observations indicate that although anti-4-1BB-pre-activated PDCA-1^+^ B cells express high levels of MHC II and take up Ag normally, they inhibit Ag-specific T cell responses.

### Anti-4-1BB pre-activated PDCA-1^+^ B cells inhibit humoral responses

**Figure 6:**
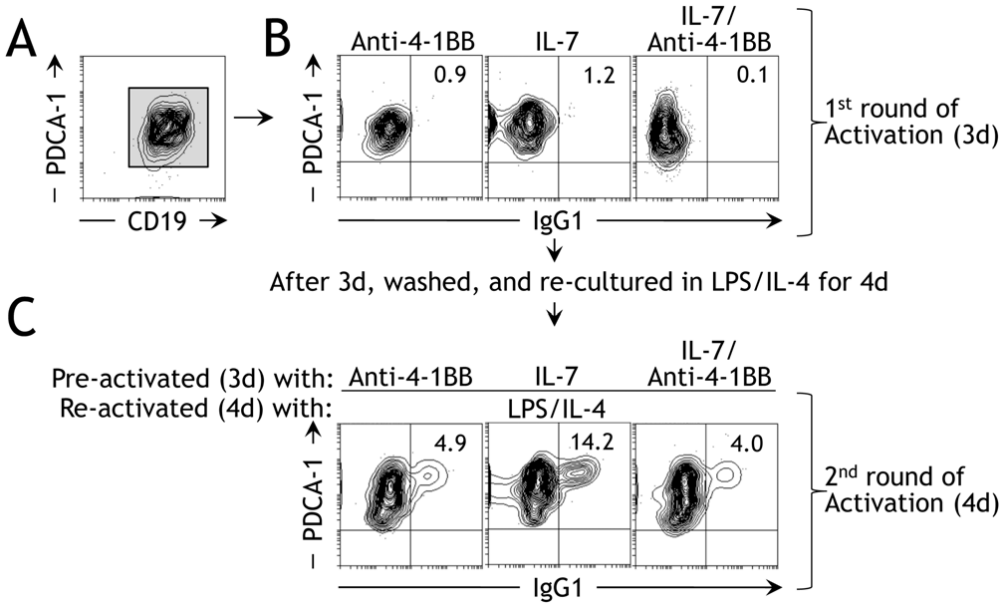
Anti-4-1BB pre-activated PDCA-1^+^ B cells inhibit in vitro Ig class switching. **A** PDCA-1^+^ B cells were sort-purified from spleens of naïve B6 mice and stained as indicated, and flow cytometry was performed. One of four independent experiments is shown (*n* = 4). **B** Purified splenic PDCA-1^+^ B cells were stimulated with the indicated agonists, after which in vitro Ig class switching was analyzed by flow cytometry. One of four independent experiments is shown (*n* = 4). **C** Purified splenic PDCA-1^+^ B cells were stimulated with soluble anti-4-1BB (5 µg/ml) and/or IL-7 (100 ng/ml) for 3d then re-stimulated with LPS/IL-4 for 4d, after which in vitro Ig class switching was analyzed by flow cytometry. One of four independent experiments is shown (*n* = 4).

Since anti-4-1BB-pre-activated PDCA-1^+^ B cells were found to inhibit Ag-specific T cell responses, we suspected that pre-conditioning with anti-4-1BB somehow altered the function and phenotype of PDCA-1^+^ B cells. To determine if this was the case, we treated purified PDCA-1^+^ B cells (Fig. 6A) with anti-4-1BB for 3d, prior to stimulation with LPS/IL-4 and studied in vitro Ig class switching. IL-7 was used as a control for the same reasons as mentioned previously [31]–[33]. We found that 3d stimulation with anti-4-1BB (Fig. 6B; left panel), IL-7 (Fig. 6B; middle panel) or both (Fig. 6B; right panel) did not induce Ig class switching, indicating that these agents do not have any direct capacity to induce Ig class switching in vitro. Interestingly, when anti-4-1BB-pre-activated PDCA-1^+^ B cells were reactivated by an IgG1-inducing LPS/IL-4 regimen, we found only 4.9% IgG1 expression (Fig. 6C; left panel). In contrast, 14.2% of PDCA-1^+^ B cells that were pre-activated with IL-7 and then reactivated with LPS/IL-4 expressed IgG1 (Fig. 6C; middle panel). When cells treated with both anti-4-1-BB and IL-7 were reactivated with LPS/IL-4, the effect of anti-4-1BB dominated over the effect of IL-7 and Ig class switching was inhibited (Fig. 6C, right panel). These data demonstrate that activation of PDCA-1^+^ B cells with anti-4-1BB inhibits in vitro Ig class switching.

**Figure 7:**
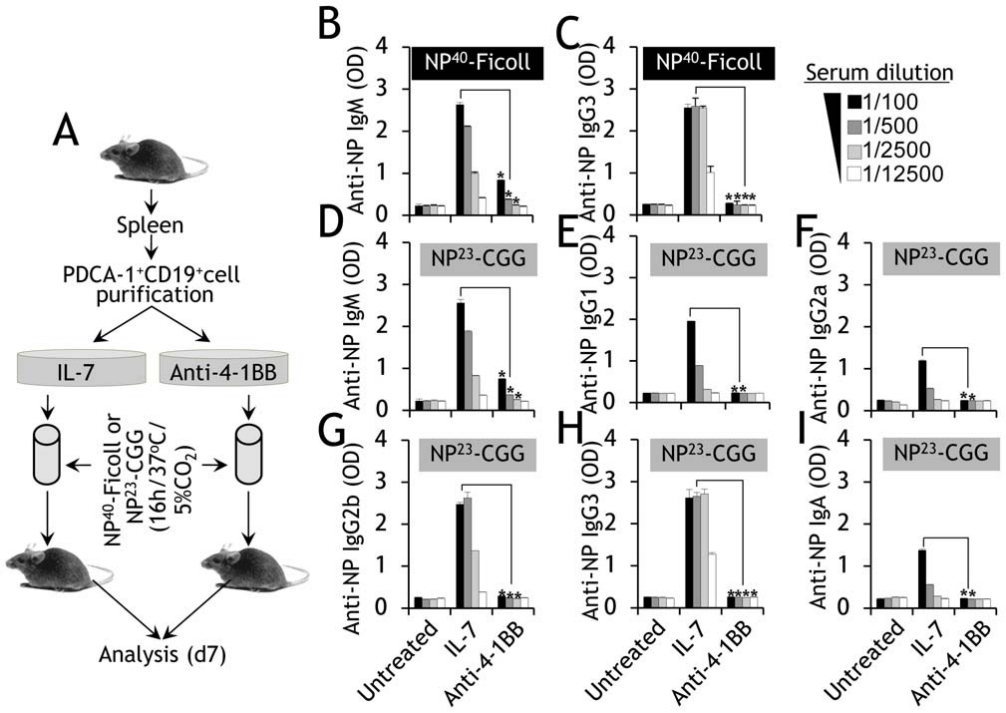
Anti-4-1BB pre-activated PDCA-1^+^ B cells inhibit in vivo humoral responses. **A** Cartoon showing the steps involved in purification of PDCA-1^+^ B cells, activation with IL-7 or anti-4-1BB, adoptive transfer, and serum analysis. **B**, **C**, PDCA-1^+^ B cells were purified from spleens of naïve B6 mice, cultured for 3d with IL-7 (100 ng/ml) or soluble anti-4-1BB (5 µg/ml), and pulsed with 0.5 mg/ml NP^40^-AECM Ficoll overnight. Ag-pulsed cells (2×10^6^) were injected (i.v.) into syngeneic hosts. Sera collected on d7 were analyzed for the presence of anti-NP IgM **B**, and IgG3 **C** Abs by ELISA. The extent of anti-NP Ab production (absorbance) is shown by the bar graphs (mean ± SD). The results of four pooled independent experiments are shown (*n* = 4). (D–I) PDCA-1^+^ B cells were purified from the spleens of naïve B6 mice, cultured for 3d with IL-7 (100 ng/ml) or soluble anti-4-1BB (5 µg/ml), pulsed overnight with 0.5 mg/ml NP^23^-CGG and adoptively transferred (2×10^6^; i.v.) into syngeneic hosts. Sera collected on d7 were analyzed for the presence of anti-NP IgM **D**, IgG1 **E**, IgG2a **F**, IgG2b **G**, IgG3 **H**, and IgA **I** Abs by ELISA. The extent of anti-NP Ab production (absorbance) is shown by the bar graphs (mean ± SD). The results of four pooled independent experiments are shown (*n* = 4). * *p*<0.05.

We next tested whether this inhibitory effect was also present in vivo. Splenic PDCA-1^+^ B cells were purified and cultured in anti-4-1BB or IL-7 for 3d. They were then washed, pulsed with NP-Ficoll (a T-independent Ag) or NP^23^-CGG (NP-CGG; a T-dependent Ag), and adoptively transferred into syngeneic recipients (Fig. 7A). The IL-7-pre-activated and NP-Ficoll-loaded PDCA-1^+^ B cells produced the expected levels of anti-NP IgM and IgG3 titers in the recipient mice, but only background titers were observed in mice that received similarly pulsed PDCA-1^+^ B cells pre-activated with anti-4-1BB (Fig. 7B and Fig. 7C). Analysis of responses to NP-CGG, which require T cell assistance to induce Ig class switching, again revealed increased titers of anti-NP IgM (Fig. 7D), IgG1 (Fig. 7E), IgG2a (Fig. 7F), IgG2b (Fig. 7G), IgG3 (Fig. 7H), and IgA (Fig. 7I) only in the mice that received IL-7-pre-activated NP-CGG-pulsed PDCA-1^+^ B cells, not in those that received similarly pulsed PDCA-1^+^ B cells pre-activated with anti-4-1BB. These results confirm that activation with anti-4-1BB transforms PDCA-1^+^ B cells into negative immune regulators with inhibited in vivo Ag-specific T and B cell responses.

## Discussion

In this study we have shown for the first time that PDCA-1^+^ B cells (PDCA-1^+^CD19^+^IgD^+^) express 4-1BB constitutively, and expression is increased upon activation. Interestingly, conv B (PDCA-1^−^CD19^+^IgD^+^) and conv pDCs (PDCA-1^+^CD19^−^IgD^−^) cells express little or no surface 4-1BB under physiological conditions, and activation results in only a modest increase in expression. The key finding of this study is that 4-1BB expression on PDCA-1^+^ B cells is functional and is involved in negative immune regulation.

It is noteworthy that we have shown that 4-1BB signals can drive PDCA-1^+^ B cell development. Analysis revealed that anti-4-1BB strongly promotes the development of PDCA-1^+^ B cells, and to a lesser extent conv B cells and conv pDCs. Our results suggest that the developing PDCA-1^+^ B cells originate from a pro B cell precursor population. Evidence for this was the complete inhibition of PDCA-1^+^ B cell development from BM-derived cells stimulated with anti-4-1BB when B220^+^ cells were depleted. This is consistent with our previous observation that PDCA-1^+^ B cells develop from ckit^+^B220^+^ pro B cell precursors [25]. We ruled out the possibility that the developing PDCA-1^+^ B cells originated from existing PDCA-1^+^ B cells or from conv pDCs, as BM-derived cells depleted of existing PDCA-1^+^ cells continued to produce PDCA-1^+^ B cells on a par with non-depleted cultures. Whether 4-1BB promotes PDCA-1^+^ B cell development by inducing growth factor production or by another mechanism is not clear, as cytokine arrays revealed the induction of only a limited number of cytokines/chemokines and no growth factors. The reason why anti-4-1BB promotes PDCA-1^+^ B cell development and not the development of conv B cells, conv pDCs, or other cells is also not clear. It could be that the much higher expression levels of 4-1BB on PDCA-1^+^ B cells than on conv B cells or conv pDCs skew the effect of anti-4-1BB in favor of PDCA-1^+^ B cell development. Because 4-1BB signaling predominantly promoted the development of PDCA-1^+^ B cells, we focused in this study on understanding its effect on these cells. The significance of 4-1BB signaling if any in conv B cells or conv pDCs will be investigated in future research.

Surprisingly, anti-4-1BB stimulation of PDCA-1^+^ B cells resulted in upregulation of only PD-L2, B7-2, and MHC II. Since PD-L2 and the B7-2 molecules are known propagators of inhibitory and costimulatory immune responses, respectively [34], [35], this finding suggests that anti-4-1BB-activated PDCA-1^+^ B cells could be both immunostimulatory and immunosuppressive. However, the observed increased expression of IDO in PDCA-1^+^ B cells in response to anti-4-1BB suggests that anti-4-1BB-activated PDCA-1^+^ B cells are poised to act as negative immune regulators. The induction of IDO, like some other factors, is dependent on the level of IFN-γ [14], [34]. Increased IFN-γ is a signature effect of anti-4-1BB in vivo [14], [34], and has been linked to IDO upregulation in DCs [14], [36]. In contrast, the increased expression of IDO in PDCA-1^+^ B cells observed in this study is independent of IFN-γ, since our cytokine array data showed there was no upregulation of this cytokine in anti-4-1BB-supplemented PDCA-1^+^ B cell cultures. This suggests the involvement of an unknown underlying mechanism of IDO upregulation. We have also explored if the PDCA^+^4-1BB^+^ B cells have phenotypic overlaps with the certain known Bregs. Recent studies have shown that certain subtypes of B cells co-expressing CD24^hi^CD38^hi^ [29] or CD24^hi^CD27 [30] and exert immune suppression vie their production of IL-10 or TGF-β or TNF-α [29], [30], [37]. Although the anti-4-1BB activated PDCA^+^ B cells reported here co-expressed CD24, CD27, and CD38 molecules, a closer evaluation revealed no expression of suppressive phenotype i.e. CD19^+^CD24^hi^CD27 or CD19^+^CD24^hi^CD38^hi^ but a 4–5-fold higher expression of regular phenotype (CD19^+^CD24^lo^CD27 or CD19^+^CD24^lo^CD38^lo^). In addition, the anti-4-1BB stimulated PDCA^+^ B cells showed no IL-10 or TNF-α (Fig. 4E) or TGF-β (data not shown) secretion in our array data indicating that these cells do not share phenotypes with the above discussed Bregs. The significance of the observed increased expression of MCP-1, RANTES, IL-12p40/p70, and sTNFR1, and the lack of induction of IFN-α in anti-4-1BB-treated PDCA-1^+^ B cells is not currently clear. Although the cellular source has not been identified, chronic anti-4-1BB treatment in vivo increases type I IFNs whose activities have been linked to several immunological irregularities [38]. The production of MCP-1 and RANTES, which help to recruit lymphocytes [39], [40], and IL-12p40/p70, which induce Th1-type immune responses [41], [42] indirectly explains the dominant type 1 (IFN-γ) phenotype seen in anti-4-1BB-treated mice [14], [36] and supports the above assumption. The exact role of the sTNFR1 induced by anti-4-1BB in PDCA-1^+^ B cells is currently unclear, but it may serve as a negative feedback mechanism by binding the TNF-α secreted during intense in vivo anti-4-1BB activity. Further investigation is needed to clarify the mechanisms by which anti-4-1BB-activated PDCA-1^+^ B cells utilize the above activation markers and cytokines to modulate immune responses.

The upregulation of 4-1BB expression on PDCA-1^+^ B cells by various agonists was anticipated but the finding that stimulation by anti-μ also significantly upregulated 4-1BB expression on PDCA-1^+^ B cells is an important observation. This effect of anti-μ is possibly because PDCA-1^+^ B cells show BCR-dependent cell division and Ig production [25]. However, our analysis of humoral responses by anti-4-1BB-activated PDCA-1^+^ B cells yielded interesting results. The suppression of these responses in anti-4-1BB-pre-activated PDCA-1^+^ B cells is of significance. It suggests that in vitro preconditioning of PDCA-1^+^ B cells with anti-4-1BB induces pronounced phenotypic changes, such as selective expression of cell surface molecules and cytokines and the production of substantial amounts of immunosuppressive IDO, leading to transformation of the PDCA-1^+^ B cells into propagators of negative immune regulation. This inference is supported by the finding that when anti-4-1BB-preconditioned PDCA-1^+^ B cells were pulsed with TI or TD Ags and adoptively transferred into syngeneic hosts, the production of Ag-specific Abs diminished. Further evidence that anti-4-1BB-pre-treated PDCA-1^+^ B cells are inhibitory is their impairment of Ag-specific cell division in OVA-specific Va2^+^CD4^+^ T cells both in vitro and in vivo. The observed inhibitory effect of anti-4-1BB-pre-activated Ag-pulsed PDCA-1^+^ B cells on Ag-specific Ab production was not due to defects in Ag presentation or viability; these cells could take up Ag normally, and we confirmed that a significant proportion of PDCA-1^+^ B cells remained viable after 3d of in vitro pre-activation with anti-4-1BB (see Fig. 5B; left panel). In addition, we recently showed that Ag-pulsed and adoptively-transferred PDCA-1^+^ B cells induce in vivo Ig class switching and Ag-specific Ab production [25]. A possible explanation for the reduction in anti-NP Ab responses is the increased expression of IDO in PDCA-1^+^ B cells exposed to anti-4-1BB. A relationship between IDO and humoral responses has recently been established: IDO^−/−^ mice were shown to display increased basal serum IgA and IgM, and in accord with this, the addition of kynurenine or piconolic acid, two IDO-generated metabolites of tryptophan, induced B cell apoptosis and inhibited in vitro IgM production [43]. Whatever the exact mechanism, we can conclude that the impaired Ab responses to TI and TD Ags resulting from adoptive transfer of anti-4-1BB pre-treated PDCA^+^ B cells into syngeneic mice are due to the inhibitory phenotype induced by anti-4-1BB pre-treatment.

Finally, the finding that anti-4-1BB functions as a growth factor for the PDCA-1^+^ B cells is significant as it can induce both the development and activation of these cells. In addition, the ability of anti-4-1BB to induce an inhibitory phenotype in PDCA-1^+^ B cells and curb Ag-specific T and B cell responses opens new avenues for achieving immune tolerance, especially in autoimmune diseases in which autoreactive T and B cells are the main factors in determining disease severity.

## Materials and Methods

All animal experiments were approved by Tulane University and National Cancer Center Institutional Animal Care and Use Committees.

### Mice

C57BL/6 (B6) and Balb/C mice were purchased from the Charles River Laboratories (Wilmington, MA), and DO11.10 (I-A^d^; Balb/c background) mice from the Jackson Laboratories (Bar Harbor, ME).

### Cell isolation

Cells were purified using either microbeads (Miltenyi Biotec, Auburn, CA) or FACS (FACS Aria; BD Biosciences, San Diego, CA). For microbead-based isolation of PDCA-1^+^ B cells, resting B cells were first isolated using CD43 microbeads in the negative fraction, followed by positive isolation of PDCA-1^+^ cells with microbeads.

### Flow cytometry

Unless otherwise stated, all antibodies were purchased from eBioscience (San Diego, CA). FcR block (clone 2.4G2; produced in-house) was added to all mixtures used for surface staining of mouse cells. Cells were stained with the appropriate fluorochrome-labeled Abs in the presence of blocking buffer (PBS with 1% BSA, Sigma-Aldrich, St. Louis, MO), washed twice, and examined by flow cytometry using a FACS LSRII (BD Biosciences).

### Bone marrow (BM) cell cultures

BM-derived cells were obtained by flushing tibiae and femurs. After lysis of red blood cells with lysing solution (Sigma-Aldrich), the remaining cells were suspended in complete medium (CM; RPMI 1640 supplemented with 10% fetal bovine serum [FBS], antibiotics, sodium pyruvate, L-glutamine, nonessential amino acids, and 2-ME; all from Lonza, Walkersville, MD). Where indicated, the cells were stimulated with FMS-like tyrosine kinase 3 ligand (Flt3L; 200 ng/ml; Peprotech, Rocky Hill, NJ), anti-4-1BB (5 µg/ml; clone 3H3, rat IgG2a; produced in-house), LPS (10 µg/ml; Sigma-Aldrich), ODN-2395 (10 µg/ml; InvivoGen), PolyI:C (10 µg/ml; Sigma-Aldrich), TNF-α (5 ng/ml; Peprotech,), IFN-γ (5 ng/ml; Peprotech), anti-CD40 (5 µg/ml; Biolegend, San Diego, CA), anti-μ (10 µg/ml; Jackson ImmunoResearch Laboratories, West Grove, PA), or CTLA-4Ig (10 µg/ml: Bristol-Myers Squibb, Wallingford, CT).

### In vitro Ig class-switching

Sort-purified PDCA-1^+^ B cells (1×10^6^/ml) were pre-incubated with IL-7 (100 ng/ml; Peprotech), anti-4-1BB (5 µg/ml), or both in 24-well culture plates in RPMI complete medium. After 3d cells, these cultures were washed and re-stimulated with LPS (25 µg/ml: *Escherichia coli* serotype 0111.B4, Sigma-Aldrich) in combination with IL-4 (5 ng/ml: Peprotech). After 4d the cells were washed and cell surface IgG1 was analyzed by flow cytometry.

### DQ-ovalbumin uptake assay

Purified PDCA-1^+^ B cells were cultured with IL-7 (100 ng/ml; Peprotech) or anti-4-1BB (5 µg/ml) for 3d, washed three times and suspended at 1×10^6^ per 100 µL in polystyrene tubes (BD Biosciences). The cells were incubated for 60 min at 4°C or 37°C with BODIPY-conjugated DQ-ovalbumin (10 µg/ml in CM) (DQ-OVA; Invitrogen, Carlsbad, CA), a self-quenched conjugate of ovalbumin that exhibits bright green fluorescence upon proteolytic processing due to release of the dye molecule. The cells were washed three times with ice-cold PBS containing 2% FBS to remove unbound or nonspecifically associated DQ-OVA, and then washed twice with ice-cold PBS, fixed with 2% paraformaldehyde, and analyzed by flow cytometry.

### Annexin V assay

Purified PDCA^+^ B cells were stimulated with rat IgG (5 µg/ml; Jackson Immunoresearch Laboratories, West Grove, PA) or anti-4-1BB (5 µg/ml) or IL-7 (100 ng/ml; Peprotech) for 3 d. The cells were then washed and incubated for 15 min with Annexin V-FITC and analyzed by FACS LSRII (BD Biosciences).

### IDO analysis

Purified PDCA-1^+^ B cells were stimulated with anti-4-1BB (5 µg/ml) for 3d, washed, and surface stained with anti-PDCA-1-Alexa 488 and anti-CD19-PE-Cy5. After fixation and permeabilization (BD Biosciences), the cells were incubated with rabbit anti-mouse IDO (0.5 µg; Enzo Life Sciences, Plymouth Meeting, PA) followed by anti-rabbit-PE (0.5 µg; eBiosciences), and analyzed by flow cytometry.

### Cytokine array

Sort-purified PDCA-1^+^ B cells from naïve mice were cultured in CM with anti-4-1BB (5 µg/ml) or IgG (5 µg/ml; Jackson Immunoresearch Laboratories). Cell-free supernatants were collected after 3d and analyzed with a cytokine array (Mouse Cytokine Antibody Array 1; Catalogue # AAM-CYT 1–2; RayBiotech, Norcross, GA) according to the manufacturer's instructions.

### Ag pulsing and adoptive transfer

PDCA-1^+^ B cells from spleens of naïve B6 mice were purified with microbeads and cultured with IL-7 (100 ng/ml) or anti-4-1BB (5 µg/ml) for 3d, washed, and pulsed overnight with 0.5 mg/ml of NP^40^-AECM Ficoll or NP^23^-CGG (Biosearch Technologies). 2×10^6^ Ag-pulsed cells were injected (i.v.) into syngeneic wt mice. Sera were collected after 7d and used to measure anti-NP Abs using ELISA plates coated with NP^26^-BSA (10 µg/ml; Biosearch Technologies) in conjunction with anti-mouse IgA-, IgM-, IgG1-, IgG2a-, IgG2b-, and IgG3-HRP (Southern Biotech, Birmingham, AL).

### Ag-specific T cell responses

PDCA-1^+^ B cells were purified from spleens of naïve B6 mice and stimulated with IL-7 (100 ng/ml; Peprotech) or anti-4-1BB (5 µg/ml) for 3d. The cells were washed three times, resuspended in CM and incubated overnight (∼16 h) in the presence of 0.5 mg/ml OVA^323–339^ (Anaspec, Freemont, CA). Unbound peptide was removed by washing 5 times with PBS. Peptide-loaded IL-7 or anti-4-1BB-pre-activated PDCA-1^+^ B cells (2×10^6^ cells) were administered i.v. into DO11.10 mice. The mice were also treated orally with BrdU (1 mg; Sigma-Aldrich) twice a day. 3d and 1 h after the last BrdU treatment, spleens were collected, stained with an FITC BrdU Flow kit (BD Biosciences) in conjunction with anti-Vα2-PE and anti-CD4-PE-Cy5, and analyzed by flow cytometry. In separate experiments, CD4^+^ T cells were negatively purified from naïve DO11.10 mice using a cell enrichment kit (Invitrogen), labeled with CFSE (10 µM; Invitrogen), and cultured for 3d in the presence of OVA^323–339^-pulsed IL-7 or anti-4-1BB-pre-activated PDCA-1^+^ B cells. The cells were washed and clumps were dissociated with EDTA (10 mM EDTA/PBS; 10 min; 37°C). The cells were then stained with anti-Vα2-PE and the extent of cell division in each sample was analyzed by flow cytometry.

### ELISA

IFN-α levels were measured in cell-free supernatants using commercially available ELISA kits (PBL InterferonSource, Piscataway, NJ) according to the manufacturer's instructions.

### Statistical analysis

Statistical significance was calculated with Student's ‘*t*’ test in Excel Software.
